# College of Physicians and Surgeons

**Published:** 1883-04

**Authors:** 


### ARTICLE XI.

### COLLEGE OF PHYSICIANS AND SURGEONS.

The first annual commencement of the College of Physicians and Surgeons of Chicago was held in the afternoon, at Central Music Hall. The attendance was quite large, and the exercises proved interesting. A fine orchestra was present and furnished some excellent music. Considering the fact that the college has completed only its first season, the number of graduates—fifty-two—speaks well for the estimation in which the Faculty is held. The members of the Faculty were all seated upon the stage, and are as follows: Profs. Bockius, Jelks, Hoadley, French, Harlan, McCoy, Silon, Gibson, MacWilliams, John, Harper, Angear, Carpenter, King, Holroyd, Waxham, Earle, Kea, Curtis, Palmer, St. John, Steele, Jackson and Powers. The exercises were opened with prayer by the Rev. Dr. Abbot E. Kittredge, of the Third Presbyterian church. After the prayer, Dr. D. A. K. Steele, secretary of the college, presented the first annual report. The college has been in existence less than two years, but during that short time it has made an enviable reputation. The first steps toward founding the college were taken by Prof. Earle, who spoke of the matter to Dr. A. Reeves Jackson. A preliminary meeting of the founders was held at the Grand Pacific hotel, May 4, 1881, and on July 2 letters of incorporation were obtained. The final certificate of incorporation was received Oct. 14, 1881, and in September of the next year the college was opened to students, and the first lecture course began in one of the finest and best appointed medical college buildings in the West. The session just closed was attended by 165 students, 52 of whom graduated yesterday.

When the secretary had finished reading his report, Dr. Jackson, President of the college, presented the diplomas, the graduates walking upon the stage in sections and receiving their passports into the field of medicine. In presenting the diplomas, Dr. Jackson said: "By virtue of the authority vested in me by the board of trustees of the College of Physicians and Surgeons of Chicago, I confer upon each of you the degree of Doctor of Medicine. I present this diploma in testimony of your conduct in having obeyed the rules and regulations of the college."

The graduates resumed their seats, and Dr. Jackson, addressing them feelingly, said:

"Gentlemen: You are the first-born of our house, and we feel the same interest in you that parents do in their first children. We have to-day publicly acknowledged you as our offspring, and we hope you will prove worthy of your parentage. This diploma is presented as a badge of your ancestry, and we trust you will never do aught to lessen the confidence we have placed in you. May God bless you in whatever you do for the right."

A number of the students were then made the recipients of floral remembrances from their lady friends. The orchestra rendered seleetions from the "Mascotte" and the class valedictorian, Dr. Orin P. Maxson, M.D., L.L.B., of Waukegan, was then introduced. Dr. Maxson delivered an eloquent farewell to his fellow-students and the Faculty, and paid a high tribute to the profession he now enters upon. He gave a synoptical history of medicine from the days of Hippocrates to the time when it attained rank as a science.

The Faculty address was delivered by Prof. A. M. Carpenter. It was a lengthy tribute to medicine, and replete with suggestions to those who were about to enter upon its practice. He urged the graduates to be careful of their professional deportment, and to so labor in the interests of the healing art as to cover themselves with renown, and reflect credit upon their Alma Mater.

The Hutton prize of $20 in gold for the best examination upon mental and nervous diseases was awarded to Theodore Henry Spencer, of Belmont, Ont.

A banquet was given by the college in the evening at the Sherman House. Some two hundred were present, composed of the members of the Faculty, graduates, and friends of the college. A band of music was stationed in the corridor at the entrance to the dining-room, and while the feast was enjoyed some very pleasant music was listened to. The tables were decked with flowers in great profusion, and three hours or more were spent in listening to addresses, recitations, and after-dinner speeches appropriate to the occasion. It was near midnight when the festivities were over.

